# Molecular Characterization and Transcriptional Regulation Analysis of the Bovine *PDHB* Gene

**DOI:** 10.1371/journal.pone.0157445

**Published:** 2016-07-05

**Authors:** Anning Li, Yaran Zhang, Zhidong Zhao, Mingming Wang, Linsen Zan

**Affiliations:** 1 College of Animal Science and Technology, Northwest A&F University, Yangling 712100, Shaanxi, P. R. China; 2 National Beef Cattle Improvement Center, Northwest A&F University, Yangling 712100, Shaanxi, P. R. China; University of Massachusetts Medical, UNITED STATES

## Abstract

The *pyruvate dehydrogenase beta subunit* (*PDHB*) is a subunit of pyruvate dehydrogenase (E1), which catalyzes pyruvate into acetyl-CoA and provides a linkage between the tricarboxylic acid cycle (TCA) and the glycolysis pathway. Previous studies demonstrated *PDHB* to be positively related to the intramuscular fat (IMF) content. However, the transcriptional regulation of *PDHB* remains unclear. In our present study, the cDNA of bovine *PDHB* was cloned and the genomic structure was analyzed. The phylogenetic tree showed bovine PDHB to be closely related to goat and sheep, and least related to chicken. Spatial expression pattern analysis revealed the products of bovine *PDHB* to be widely expressed with the highest level in the fat of testis. To understand the transcriptional regulation of bovine *PDHB*, 1899 base pairs (bp) of the 5’-regulatory region was cloned. Sequence analysis neither found consensus TATA-box nor CCAAT-box in the 5’-flanking region of bovine *PDHB*. However, a CpG island was predicted from nucleotides -284 to +117. Serial deletion constructs of the 5’-flanking region, evaluated in dual-luciferase reporter assay, revealed the core promoter to be located 490bp upstream from the transcription initiation site (+1). Electrophoretic mobility shift assay (EMSA) and chromatin immunoprecipitation assay (ChIP) in combination with asite-directed mutation experiment indicated both myogenin (MYOG) and the CCAAT/enhancer-binding protein beta (C/EBPß) to be important transcription factors for bovine *PDHB* in skeletal muscle cells and adipocytes. Our results provide an important basis for further investigation of the bovine *PDHB* function and regulation in cattle.

## Introduction

The pyruvate dehydrogenase complex (PDC) is a “gateway” enzyme for the mitochondrial oxidative metabolism of carbohydrates, catalyzing pyruvate into acetyl-CoA and carbon dioxide, and providing a linkage between glycolysis and the tricarboxylic acid cycle (TCA) pathway [1]. PDC is composed of three enzymatic components: pyruvate dehydrogenase (E1), dihydrolipoamide acetyltransferase (E2) and lipoamide dehydrogenase (E3). PDC plays a central role in the maintenance of glucose homoeostasis in mammals [2]. The activity of PDC is regulated by phosphorylation and dephosphorylation [1] and SIRT4 [3]. Moreover, PDC deficiency is associated with various diseases, such as epilepsy [4,5], primary lactic acidosis [6], and cardiovascular diseases [7].

The enzyme E1 is a heterotetramer consisting of two alpha and two beta subunits which are encoded by the *pyruvate dehydrogenase alpha subunit* (*PDHA*) and the *pyruvate dehydrogenase beta subunit* (*PDHB*), respectively. The product of *PDHB* expression is distributed in mitochondria [8]. Mutations in the *PDHB* gene lead to a series of metabolic diseases, such as primary lactic acidosis [9], leigh syndrome [10], and congenital deficiency of the PDC [11]. Moreover, in gastric cancer cells, over-expression of *PDHB* helps the pyruvate metabolism to move into the TCA process rather than into the glycolysis process, consequently inhibiting cell growth and cell migration [12]. In addition, PDHB was identified to interact with prolyl-hydroxylase PHD3 [13] and ATP6AP2 [14].

The intramuscular fat (IMF) content is one of the most important traits for meat quality affecting flavor, juiciness and tenderness of meat [15,16]. Recent studies show the positive relationship between the *PDHB* expression level and the IMF content. Furthermore, *PDHB* expression was reported to be significantly different between low-marbled and high-marbled steer groups in beef cattle [17]. In pigs, the expression level of *PDHB* is positively related to the IMF content in the longissimus dorsi muscle [18]. Similarly, *PDHB* expression is significantly different between lean and fat lines of the Pekin duck [19]. Furthermore, using a quantitative proteomics approach, our previous study revealed the protein expression level of *PDHB* to be significantly different between obese and lean pigs [20]. Therefore, we speculate the *PDHB* gene to play an important role in animal IMF deposition.

However, the published information on the bovine *PDHB* gene is very limited. In this study, the full length cDNA was cloned and the spatial expression pattern of bovine *PDHB* was analyzed. In addition, the transcriptional activity of the bovine *PDHB* gene 5’-regulatory region was identified in both skeletal muscle cells and adipocytes. Our results provide a solid basis for further research on the functions of the bovine *PDHB* gene.

## Materials and Methods

### Ethics Statement

All animal procedures were performed according to guidelines laid down by the China Council on Animal Care, and the protocols were approved by the Experimental Animal Manage Committee (EAMC) of Northwest A&F University.

### Molecular cloning and sequence analysis

The complete *PDHB* coding sequence (CDS) with primers (*PDHB*-CDSF/R, Table 1) and longissimus dorsi muscle cDNA was cloned. To obtain the 5’-regulatory region, we searched the *PDHB* gene in the UCSC genome database (http://genome.ucsc.edu/cgi-bin/hgGateway). We isolated a bovine genomic sequence (bosTau4_refGene_NM_001035435 range = chr22:43767058–43774471) and cloned 1899 bp of 5’-regulatory region sequence with the primers (*PDHB*-PF/R, Table 1). We used genomic DNA extracted from Qinchuan cattle blood as template and applied 2×GC Buffer I (TaKaRa, Biotechnology Co. Ltd, Dalian, P.R.C.) to amplify 5’-regulatory region sequence. The potential transcription factor binding sites were analyzed using the Genomatix suite (http://www.genomatix.de/), TESS (http://www.cbil.upenn.edu/cgi-bin/tess/tess?RQ=WELCOME) and TFSEARCH (http://www.cbrc.jp/research/db/TFSEARCH.html). CpG islands were predicted using Meth Primer (http://www.urogene.org/methprimer/).

**Table 1:** Primers utilized in this study.

Primer name	Primer sequence(5’-3’)	Binding region	PCR(TM) (°C)	Size (bp)
*PDHB*-CDSF	AGATGGCGGTGGTTGCTGTG	5’-UTR	63	1166
*PDHB*-CDSR	ATTAAAAGGTCTTATGGGAT	3’-UTR	—	—
*PDHB*-PF	CCCAATTTCTTGCATCTCACCAT	-1724/-1702	60.9	1899
*PDHB*-PR	CTGGCAGGGAGAGAGAGAAGACGC	+152/+175	—	—
*PDHB*-GSP1	CACTGGGTTGTTATCCCGAATGGC	+659/+682	68	682
*PDHB*-GSP2	CCCAGCCATAGCGGCACCTACAGC	+377/+400	60	400
*GAPDH*-F	CCAACGTGTCTGTTGTGGAT	—	60	80
*GAPDH*-R	CTGCTTCACCACCTTCTTGA	—	—	—
*PDHB*-RT-F	TCTGAGATGGGCTTTGCTGG	Exon 3–4	60	109
*PDHB*-RT-R	TGACCTGGTCGATGGCTTGC	Exon 5	—	—
*PDHB*-R	***CCCGGG***TTCCGCACCAACACAGC	+103/+119	—	—
*PDHB*-P1	***GGTACC***CCCAATTTCTTGCATCT	-1725/-1709	60	1844
*PDHB*-P2	***GGTACC***TTTCTCTCTCTAGCACT	-1354/-1338	60	1473
*PDHB*-P3	***GGTACC***GGCCTCCCTGCCTCTGC	-1054/-1038	60	1173
*PDHB*-P4	***GGTACC***TCACTAGGCGTGTCCGA	-790/-774	60	909
*PDHB*-P5	***GGTACC***TTCACTCTATCCATCTG	-490/-474	60	609
*PDHB*-P6	***GGTACC***CCAAAATACCAACACGG	-190/-174	60	309
MYOG-F	TTAACATAA***TGCCTGG***CACACATGAAA	-388/-382	—	609
MYOG-R	TTTCATGTGTG***CCAGGCA***TTATGTTAA	—	—	—
m MYOG-F	CTTAACATAATGC***TGC***GCACACATGAAAGC	-388/-382	60	609
m MYOG-R	GCTTTCATGTGTGC***GCA***GCATTATGTTAAG	—	—	—
C/EBPß-F	AAAGACAGTG***TTTCCAG***GGTAACTTGT	-331/-325	—	609
C/EBPß-R	ACAAGTTACC***CTGGAAA***CACTGTCTTT	—	—	—
mC/EBPß-F	CCAAAAGACAGTGT***GGA***CAGGGTAACTTGTCAG	-331/-325	60	609
mC/EBPß-R	CTGACAAGTTACCCTG***TCC***ACACTGTCTTTTGG	—	—	—
ChIP- MYOG-F	GCACTTAACATAATGCCTGG	-488/-463	60	168
ChIP- MYOG-R	ACATAATCTGGTGACTGGGT	-340/-321	—	—
ChIP-C/EBPß -F	CCTATGAGAATAAAGTGAGC	-418/-399	60	120
ChIP-C/EBPß -R	AGTCTGACAAGTTACCCTGG	-318/-299	—	—
ChIP-control-F	GATGTGCCTATGCCTTATGC	Exon 10	60	125
ChIP-control-R	ATTCCAATGACCTGAACTTC	Exon 10	—	—

We obtained the 4kb5'-regulatory region of *PDHB* in cattle (gi|258513345:43457246–43461246), sheep (gi|417531901:42930424–42934424), goat (gi|541128965:42734750–42738750), porcine (gi|347618781:c44131745-44127745), dog (gi|357579611:32181538–32185538), human (gi|568815595:c58437807-58433807), mouse (gi|372099096:c8176930-8172930), rat (gi|666183559:18537242–18541242) and chicken (gi|358485500:c11816992-11812992) from the genome database at the National Center for Biotechnology Information (NCBI). We performed multi-alignments of the 5'-regulatory region among the nine species using ClustalX 2.0.

### Structural and phylogenetic tree analysis

We used the SMART database (http://smart.embl-heidelberg.de/) to predict putative domains. We constructed the phylogenetic tree as previously described [21].

### 5’-Rapid amplification of cDNA ends (5’-RACE)

To identify the transcription initiation sites, 5’-RACE from total RNA of subcutaneous fat was performed according to the manufacturer’s protocol using the SMART^TM^ RACE Kit (Clontech Inc, Palo Alto, CA, USA). We applied the nest PCR primers (*PDHB*-GSP1 and *PDHB*-GSP2, Table 1) to obtain the 5’-end of the *PDHB* gene and used touchdown PCR in the first PCR with conditions as previously described [21]. The second PCR template was a 20-fold dilution of the first PCR products.

### Real-time PCR analysis of spatial expression pattern

We obtained sixteen tissue samples (longissimus dorsi muscle, kidney, heart, biceps femoris, liver, subcutaneous fat, spleen, lung, cecum, rumen, reticulum, omasum, abomasums, testis fat, large intestine and small intestine) from three adult Chinese indigenous Qinchuan cattle and selected *glyceraldehyde-3-phosphate dehydrogenase* (*GAPDH*) as the endogenous reference. We used the primers of *PDHB*-RT-F/R (Table 1) and *GAPDH*-F/R (Table 1) [22] for this assay. The cycling conditions were according to SYBR Premix Ex Taq^TM^Ⅱ (TaKaRa, Biotechnology Co. Ltd, Dalian, P.R.C.), using the ABI 7500 RT-PCR system (Applied Biosystems, USA). We used the 2^-△△Ct^ method for this assay.

### Cell culture, transfection and dual-luciferase reporter assay

We cultured the mouse myoblast cell line (C2C12) and the 3T3-L1 cell line in Dulbecco's modified Eagle's medium (DMEM) supplemented with 10% fetal bovine serum (FBS) (GIBCO-Invitrogen) under humidified air containing 5% CO2 at 37°C. 5’-nested PCR primers (*PDHB*-P1~P6, Table 1) and common 3’ primer (*PDHB*-R, Table 1) were used to amplify six serial deletion fragments of the 5’-flanking region. Then these fragments were cloned into the *Kpn*I-*Sma*I site of the pGL3-basic vector (Promega Corp.). After sequencing verification, we extracted the plasmids with an Endo-free Plasmid Mini Kit (OMEGA, USA). We co-transfected the plasmids (0.8 μg) and the lipofectamine 2000 (2μl) into C2C12 or 3T3-L1 cells grown in 24-well plates with 10 ng pRL-TK (Promega Corp.). At 5 h after transfection, we replaced the media with DMEM with 2% horse serum (HS) (GIBCO-Invitrogen) and incubated for 40 h to induce differentiation of the C2C12 myoblasts into myotubes. We performed all remaining steps as previously described [21].

### Site-directed mutagenesis

We mutated the transcription factor-binding sites for MYOG and C/EBPß with the corresponding primers (mMYOG-F/R, mC/EBPß-F/R, Table 1) using the Quick Change Site-Directed Mutagenesis Kit (Stratagene, La Jolla, CA, USA). PCR was carried out as previously described [21].

### Electrophoretic mobility shift assay (EMSA)

After differentiation of the C2C12 myoblasts into myotubes for 7 days, we prepared nuclear protein extracts as previously described [21,23]. We incubated probes of a 200 fmol of 5'-biotin labeled MYOG (or C/EBPß) with 10μg nuclear extracts of C2C12 myotubes (or 10μg3T3-L1nuclear extracts), 2μl 10×binding buffer, 1μl 50% Glycerol, 1μl MgCl2 and 1μl poly (dI.dC) in a volume of 20 μl. We incubated 5μg of antibodies myogenin (sc-12732×, Santa Cruz Biotechology, CA) (or C/EBPß (sc-150×, Santa Cruz Biotechology, CA)) with the nuclear extracts before adding the labeled probes. We used 6% polyacrylamide gels to separate DNA-protein complexes. Several steps followed according to the Light Shift® Chemiluminescent EMSA Kit (Pierce Corp., Rockford, IL, USA) Manufacturer’s Instructions.

### Chromatin immunoprecipitation (ChIP) assay

We isolated longissimus dorsi muscle and inguinal fat from bulls at 3 days after birth (n = 3). We conducted the ChIP assay using the EZ-ChIP™ Kit (Millipore, Bedford, MA, USA) according to the manufacturer’s protocol. We cross-linked the DNA-protein complexes with 37% formaldehyde and neutralized with glycine. After sonication, we added about 200–1000 bp of fragmented chromatin into the ChIP dilution buffer. We immunoprecipitated an equal amount of chromatin overnight at 4°C with 4μg of the myogenin (or C/EBPß) antibodies and normal mouse IgG. We then collected the immunoprecipitated products with Protein A+G coated magnetic beads. We eluted the bound chromatin in ChIP Elution Buffer and digested with proteinase K, then purified the DNA for PCR analysis. We used the primers (ChIP-MYOG-F/R and ChIP-C/EBPß-F/R, Table 1) and the negative control primers (ChIP-control-F/R, Table 1) in the RT-PCR and ChIP-QPCR. % Input = 2^(-△Ct (Ct [ChIP]—(Ct [Input]—Log2 (Input Dilution Factor))))^ [24]. As a negative control, we used the immunoprecipitate products from the normal mouse IgG group.

### Statistical analysis

All values are expressed as mean±standard deviation (SD). We analyzed the differences between groups with a Student’s two-tailed T-test. * P<0.05. ** P<0.01. n = 3.

## Results

### Molecular cloning and sequence analysis

**Fig 1:**
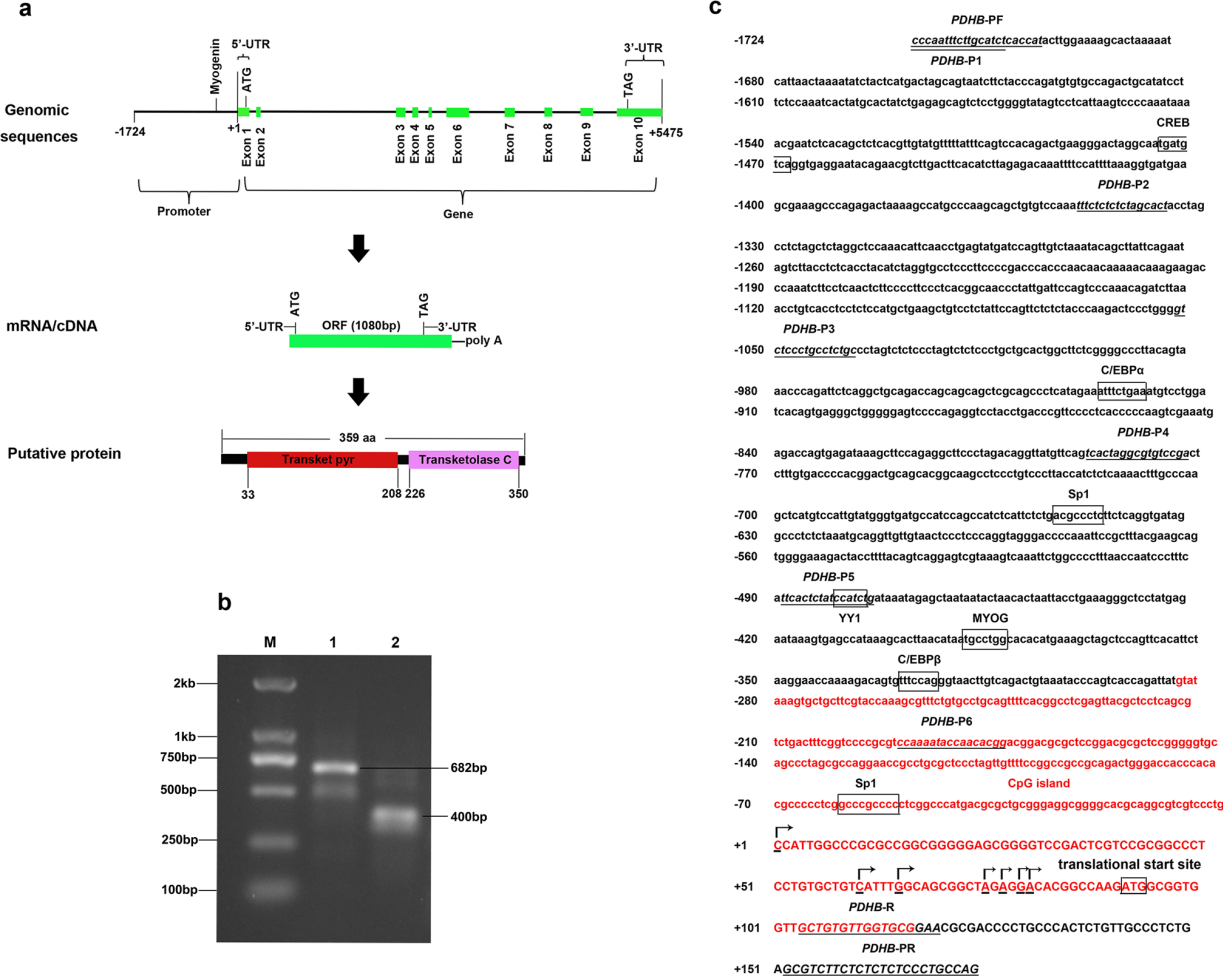
Structure characteristics of the bovine *PDHB* gene. a. Here we show the genomic, mRNA and protein components in detail. 5’- UTR:5’- untranslated region, 3’- UTR:3’- untranslated region, ORF: open reading frame, Transketpyr: transketolase, pyrimidine binding domain. b. 5’-RACE. Lane 1 and 2 are products of the first and second PCR, respectively. Lane M represents the marker of DL2000. c. 5’-regulatory region sequence of bovine *PDHB* gene. Arrows mark the transcription initiation sites. The cytosine residue is designated as +1. The transcription factor binding sites are boxed. The primers are underlined with the respective names below the line. The CpG island is indicated with red color.

Based on the bovine *PDHB*c DNA sequence (GenBank No. NM_001035435.3), we obtained a1576 bp cDNA. It contained an open reading frame (ORF) of 1080 bp, which had 359 amino acids (aa) encoded with a calculated molecular mass of 39.13 kDa and an isoelectric point (pI) of 6.466. Our cDNA consisted of a 5’-UTR of 91 bp and a 3’-UTR of 405 bp with a consensus AATAAA polyadenylation signal 16 bp upstream of the poly (A) stretch. The bovine *PDHB* gene spanned approximately 5.49 kb on the genome, containing 10 exons and 9 introns (Fig 1A). In addition, the bovine PDHB protein contains two putative domains: the transketolase, pyrimidine binding domain (Transketpyr) resides in 33-208 amino acid residues and the transketolaseC domain resides in 226–350 aa (Fig 1A).

**Fig 2:**
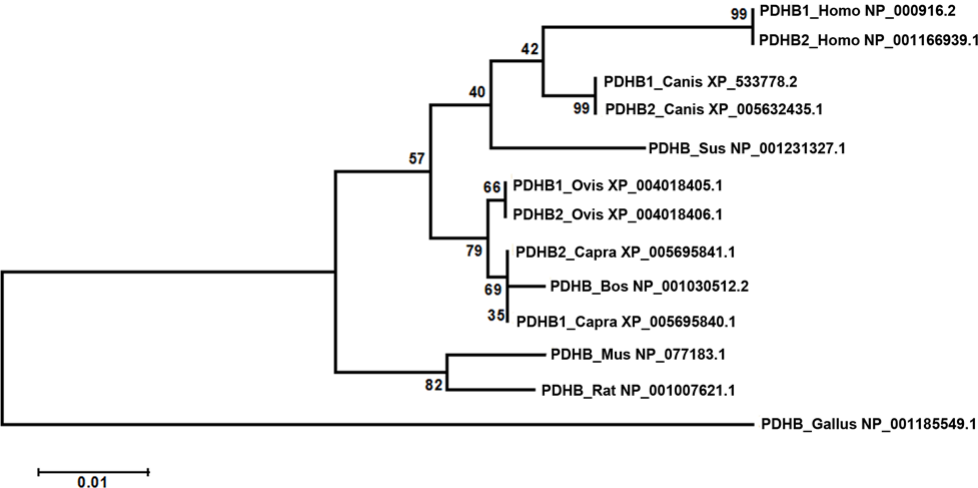
Phylogenetic tree analysis of PDHB. We calculated 8000 bootstrap replicates to bootstrap confidence values.

The bovine PDHB amino acid sequence shared a high similarity with other mammalian, with the following levels of identity: sheep PDHB1 (99%), goat PDHB1 (99%), porcine (97%), dog PDHB1 (96%), sheep PDHB2 (94%), goat PDHB2 (94%), mouse (94%), rat (94%), human PDHB1 (94%), human PDHB2 (89%), dog PDHB2 (86%) and chicken (82%). The phylogenetic tree indicated the bovine PDHB to be closest related to goat and sheep, and the lowest relatedness to chicken of all nine species evaluated for this study (Fig 2).

### Transcription initiation site of the bovine PDHB gene

In the first and second PCR, we cloned 682 bp and 400 bp fragments, respectively (Fig 1B). Sequencing eleven positive clones identified seven different 5’ ends with 91 bp, 31 bp, 26 bp, 16 bp, 14 bp, 12 bp and 11 bp upstream from the translational start site, respectively (Fig 1C). We designated the cytosine residue (C) as +1 and found it to be located 91 bp upstream from the translational start site.

### Characterization of the bovine PDHB gene 5’-regulatory region

We cloned a 1899 bp fragment of the bovine *PDHB* gene 5’-regulatory region spanning nucleotides from nucleotides -1724 to +175 and submitted it to GenBank (GenBank No. KJ649747). Several transcription factor-binding sites were recognized via sequence analysis of the 5’-regulatory region, including the Sp1 transcription factor (Sp1), the transcription factor Yin Yang-1 (YY1), the CCAAT/enhancer-binding protein ɑ (C/EBPɑ), myogenin (MYOG), the CCAAT/enhancer-binding protein ß (C/EBPß) and the cAMP responsive element-binding protein (CREB) (Fig 1C). Interestingly, we did not find a consensus TATA-box or CCAAT-box in the 5’-flanking region. However, using Meth Primer, a CpG island with a length of 401 bp between nucleotides -284 and +117 was predicted (Fig 1C).

Compared to the bovine *PDHB* gene 5’-regulatory region (GenBank No. KJ649747), the 5'-flanking sequences of cattle, goat and sheep shared 97%, 94% and 93% sequence similarity. However, it had no significant sequence similarity with the 5'-flanking sequences of porcine, dog, human, rat, mouse or chicken *PDHB*.

### Spatial expression pattern of the bovine PDHB gene

**Fig 3:**
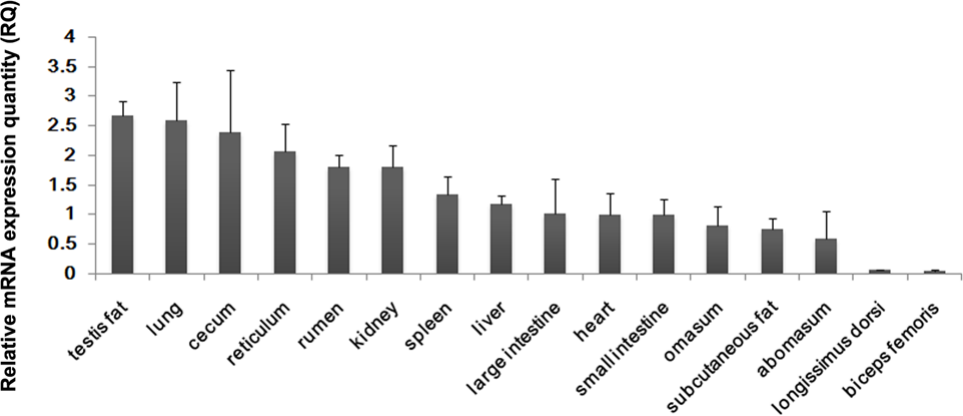
Spatial expression analysis of bovine *PDHB* mRNA. We normalized the mRNA expression levels of *PDHB* to those of *GAPDH*. Error bars represent the standard deviation (SD) (n = 3).

In order to understand the role of the bovine *PDHB* gene products in various tissues, it was necessary to provide mRNA expression profiles via real-time PCR analysis. The results showed the highest expression in testis fat, followed by lung, cecum, reticulum, rumen, kidney, spleen, liver, large intestine, heart, small intestine, omasum, subcutaneous fat, abomasums, longissimus dorsi and biceps femoris (Fig 3). This result indicates the *PDHB* mRNA to be widely expressed with the highest level in testis fat, revealing the *PDHB* gene to be a housekeeping gene in multiple tissue types.

### Transcriptional regulation of the bovine PDHB gene

**Fig 4:**
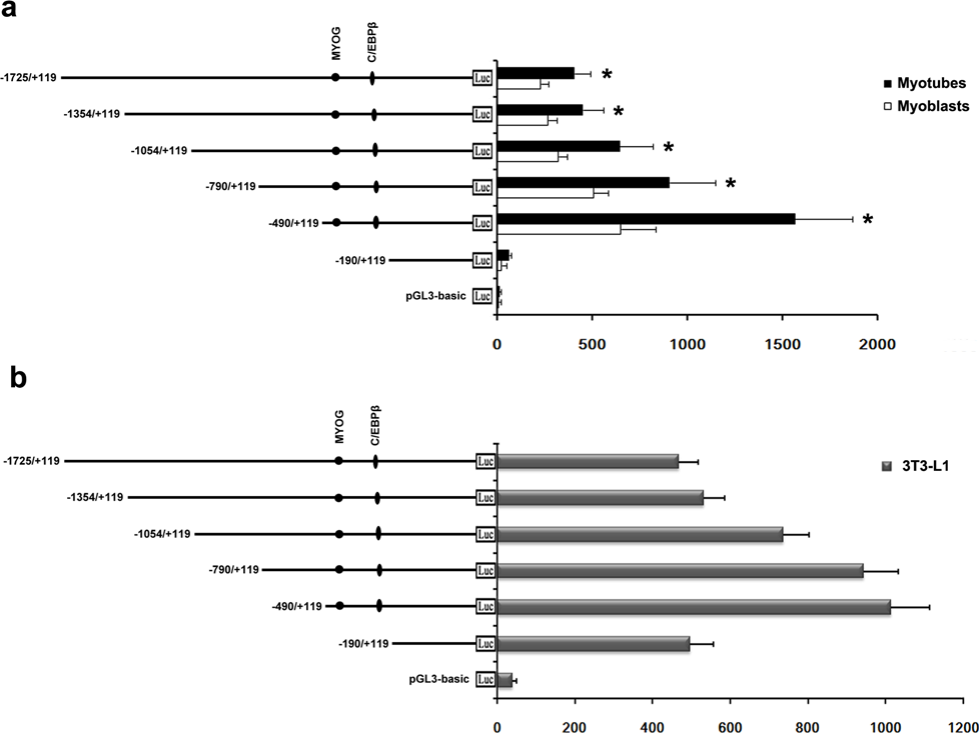
Promoter activity analysis of the bovine *PDHB* gene. a. We transferred six serial deletion constructs in pGL3-basic into C2C12 cells. After 5 h we replaced the transfection mixture with DMEM with 5% FBS (myoblasts) or 2% HS (myotubes). b. We transferred the same constructs into 3T3-L1 cells. We normalized relative luciferase activities to Renilla luciferase activity. The transcription factor binding sites of MYOG and C/EBPß are indicated with closed circles and ellipses, respectively. *, P<0.05. Error bars represent the SD (n = 3).

To identify the transcriptional activity of the bovine *PDHB* gene 5’-regulatory region, we generated six serial deletion constructs in pGL3-basic containing -1725/+119, -1354/+119, -1054/+119, -790/+119, -490/+119 and -190/+119. After we transfected the construct plasmids into C2C12 cells, the transcriptional activity of the construct -490/+119 was about 25-fold higher than the construct -190/+119 (Fig 4A). The activities of all other constructs were higher than the construct -190/+119 and lower than that of the construct -490/+119. Inducing the C2C12 cells via HS after transfection, significantly increased the transcriptional activities of the six constructs and the activity of the construct -490/+119 was highest in the six constructs. After transferring the construct plasmids into 3T3-L1 cells, the transcriptional activity of the construct -490/+119 was also highest in the six constructs and the activity of the construct -190/+119 was increased compared to that of the C2C12 cells (Fig 4B). These results suggest a core functional promoter to be present in the upstream region of 490 bp from the transcription initiation site.

**Fig 5:**
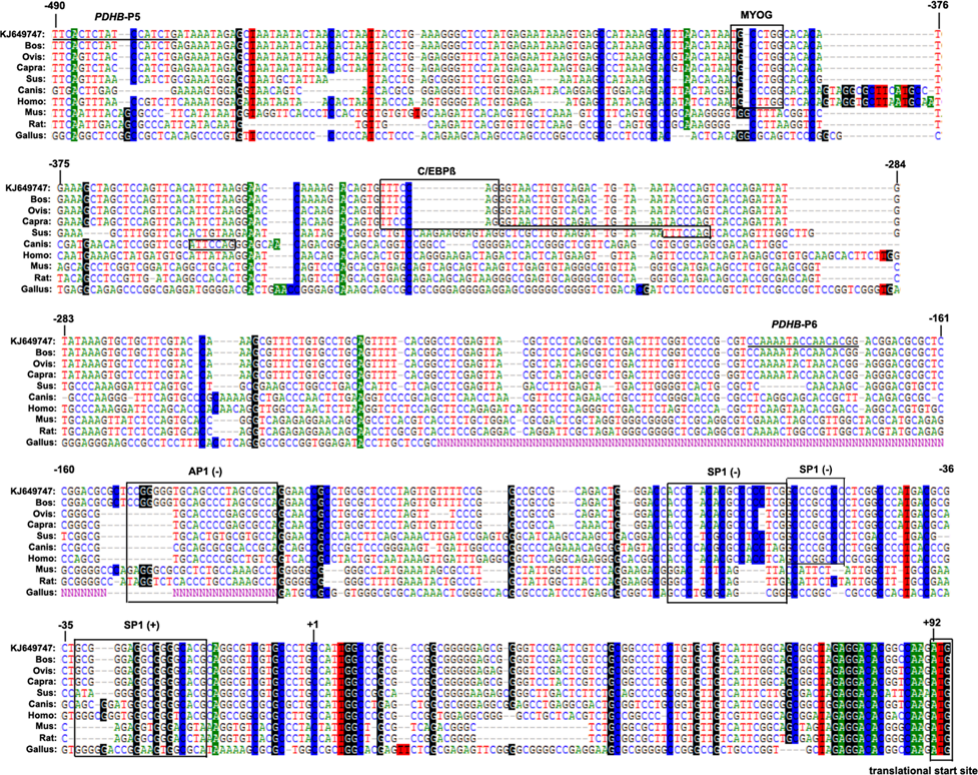
Multi-alignments sequence analysis of the core functional promoter of bovine *PDHB* in relation toother mammals. The transcription factor binding sites are marked with boxes. The nucleotide sequence is numbered in 5'-regulatory sequence of the bovine *PDHB* gene (GenBank No. KJ649747).

**Fig 6:**
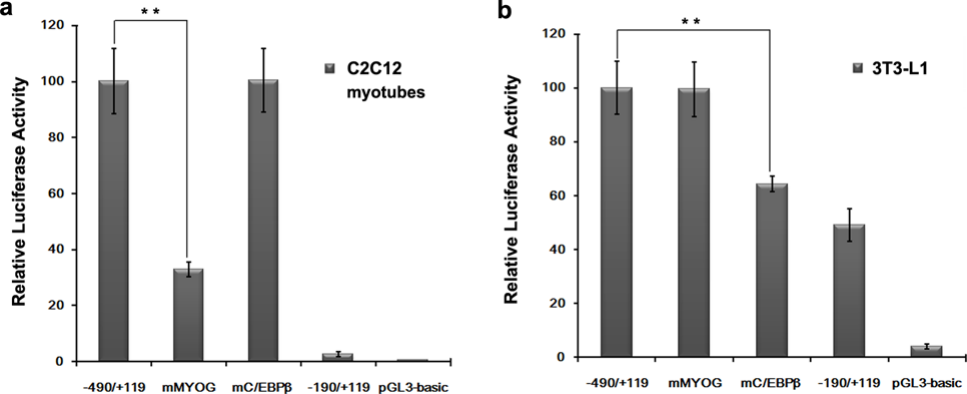
Functional analysis of the mutated MYOG and C/EBPß sites. We transferred the mutated sites MYOG and C/EBPß into C2C12 myotubes (a) and 3T3-L1 cells (b). **, P<0.01. Error bars represent the SD (n = 3).

To identify important positive regulatory elements among the nine species, the region from nucleotides -490 to +92 was predicted by transcription factor binding sites prediction software. The result of multi-alignments revealed MYOG, C/EBPß SP1 and AP1 to be conserved in this region for domestic animals such as cattle, sheep, goat, porcine and dog (Fig 5). Mutation of the MYOG site at position -388 to -382 led to a sharp decrease in activity of 70%, while mutation of the C/EBPß site at position -331 to -325 had no effect on the promoter activity of C2C12 myotubes (Fig 6A). In contrast, mutation of the C/EBPß site at position -331 to -325 led to a decrease in activity of 36%, while mutation of the MYOG site at position -388 to -382 had no significant effect on promoter activity in 3T3-L1 cells (Fig 6B).

**Fig 7:**
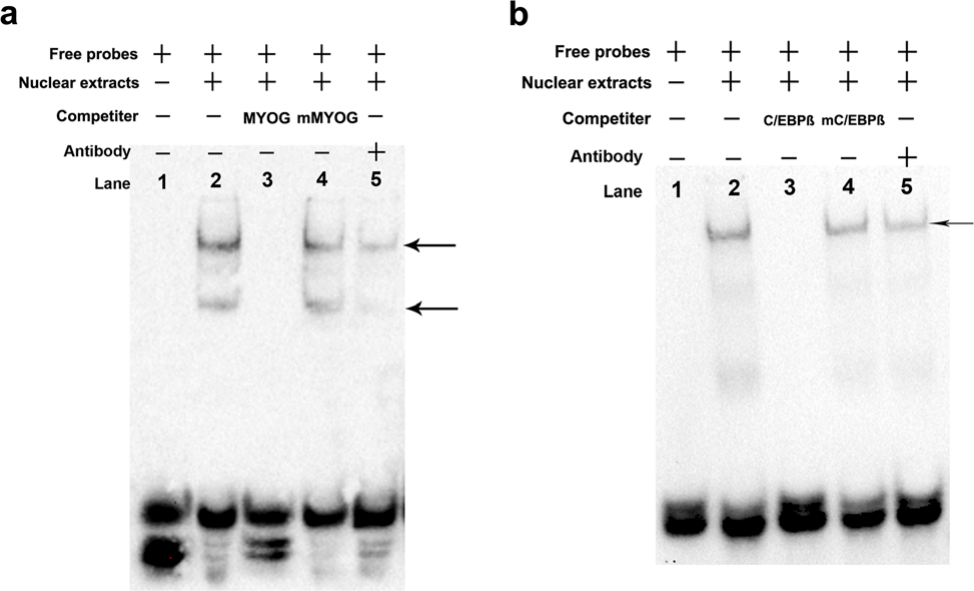
EMSA involving 5’-biotin labeled MYOG and C/EBPß probes. a. 5’-biotin labeled MYOG probes and nuclear extracts of C2C12 myotubes. Lane 1: MYOG probes; lane 2: MYOG probes with nuclear extracts of C2C12 myotubes; lane 3: MYOG probes and nuclear extracts with a 125-fold unlabeled MYOG oligonucleotides; lane 4: MYOG probes and nuclear extracts with a 125-fold unlabeled mMYOG oligonucleotides. lane 5: MYOG probes and nuclear extracts with myogenin antibodies. b. 5’-biotin labeled C/EBPß probes and nuclear extracts of 3T3-L1 cells. Lane 1: C/EBPß probes; lane 2: C/EBPß probes with nuclear extracts of 3T3-L1 cells; lane 3: C/EBPß probes and nuclear extracts with 125-fold unlabeled C/EBPß oligonucleotides; lane 4: C/EBPß probes and nuclear extracts with 125-fold unlabeled mC/EBPß oligonucleotides. lane 5: C/EBPß probes and nuclear extracts with C/EBPß antibodies.

**Fig 8:**
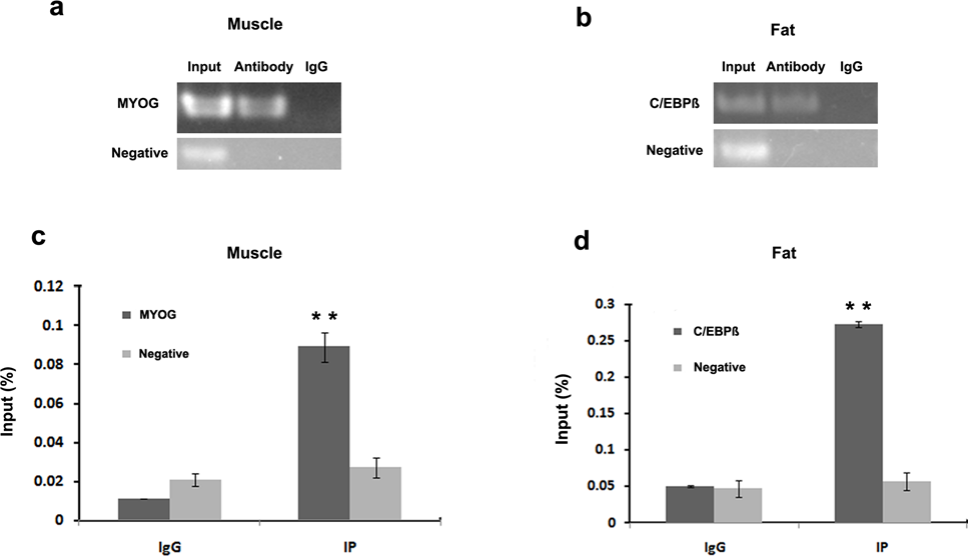
ChIP assay of MYOG and C/EBPß binding to PDHB promoter in vivo. We analyzed immunoprecipitated products for MYOG (a) and C/EBPß (b) antibodies via RT-PCR. We analyzed immunoprecipitated products for MYOG (c) and C/EBPß (d) antibodies via ChIP-QPCR. We used total chromatin from muscle (a and c) and fat (b and d) as the input. We used normal mouse IgG as the negative control antibodies. **, P<0.01. Error bars represent the SD (n = 3).

In order to validate interaction between the transcription factors MYOG and C/EBPß with the 5’-regulatory region of *PDHB*, we carried out EMSA and ChIP assays both in vitro and vivo. The EMSA results revealed that 5’-biotin labeled MYOG probes with nuclear extracts of C2C12 myotubes formed two upshifted bands (Fig 7A, lane 2). When 5’-biotin labeled MYOG probes were added with unlabeled MYOG oligonucleotides, the upshifted bands disappeared (Fig 7A, lane 3). The shifted MYOG complexes did not compete with unlabeled mutated MYOG oligonucleotides (Fig 7A, lane 4). Adding the MYOG antibody strongly diminished the upshifted bands (Fig 7A, lane 5). C/EBPß site and 3T3-L1 cells nuclear extracts exhibited similar results with MYOG site and C2C12 myotubes nuclear extracts (Fig 7B). The ChIP results revealed MYOG interacted with the MYOG binding site (Fig 8A and 8C) and C/EBPß interacted with the C/EBPß binding site in the *PDHB* promoter (Fig 8B and 8D).

## Discussion

The bovine PDHB aa sequence had 99% identity to goat and sheep, and was most closely related to goat and sheep. In addition, the bovine *PDHB* gene 5’-regulatory region shared 94% and 93% sequence similarity with goat and sheep, respectively. These results show that *PDHB* gene is highly conservative in ruminants. The bovine *PDHB* contained the two putative domains transketpyr and transketolaseC, which indicates its function to be mainly displayed in the energy metabolic process. The result of spatial expression patterns revealed the expression level of *PDHB* to be higher in fat compared to muscle, which is consistent with the expression level of *PDHB* being positively related with the IMF content [17–20]. This result implies that the *PDHB* gene could be used as a molecular marker for the IMF content.

To date, this is the first research report on transcriptional regulation analysis in the bovine *PDHB* gene. In the present study, we identified seven transcription initiation sites for the first time. Multiple transcription initiation sites are a typical feature in TATA-less promoters [21,25]. There was no alternatively spliced transcript found in the bovine *PDHB*, while two transcripts were presented in sheep, goat and human *PDHB* [26], respectively.

To understand the transcriptional regulation of the bovine *PDHB* gene, we cloned 5’-regulatory region spanning nucleotides from -1724 to +175. Sequence analysis revealed that no consensus TATA-box or CCAAT-box was present in the 5’-flanking region, which was similar to that of human *PDHB* [27]. However, GC-rich regions in the bovine *PDHB* gene promoter indicate that the transcription activity might be dependent on the methylation level of the CpG island [28]. The deletion analysis of the 5’-regulatory region showed the highest transcription activity of the construct -490/+119, while we found the transcription activity of the construct -1354/+119 to be significantly decreased in C2C12 and 3T3-L1 cells. This demonstrates the region from nucleotides -490 to +119 to be a core functional promoter and there were repressor binding sites present between -490 and-1354.

After analysis with online prediction software, we found the potential transcription factor binding sites MYOG and C/EBPß on the sequence from nucleotides -490 to -190 to be conserved in domestic animals. Myogenin (MYOG) is a member of the family of muscle regulatory factors (MRFs) that also include myogenic differentiation 1 (MYOD), myogenic factor 5 (MYF5) and myogenic regulatory factor 4 (MRF4, or MYF6) [29–31]. MYOG is a muscle-specific, basic-helix-loop-helix (bHLH) transcription factor, which is up-regulated during the differentiation of myoblasts into multinucleated myotubes [29] and essential for the development of functional skeletal muscle [31]. A recent study reported MYOG to be a positive regulator in the transcriptional regulation of *muiple EGF-like domain 10* (*MEGF10*) in C2C12 cells [32]. Mutation of the MYOG site in the present study in C2C12 myotubes, significantly decreased the transcription activity of *PDHB* promoter. Mixing 5’-biotin labeled MYOG probes with nuclear extracts of C2C12 myotubes, led to the upshift of two bands. We suspect the reason to be heterodimers-formation between MYOG and other bHLH proteins [33,34]. The ChIP assay confirms that transcription factor MYOG is capable of binding to the MYOG binding site in the *PDHB* promoter. The EMSA and ChIP results combined with mutation analysis suggest MYOG likely to be an important transcription factor regulating the expression of the bovine *PDHB* gene in skeletal muscle cells.

C/EBPβ is a member of the family of CCAAT/enhancer binding proteins (C/EBPs), and binds to promoter regions of target genes to regulate the expression of downstream genes [35]. A recent publication revealed C/EBPß to be expressed early to activate PPARγ, which is expressed in terminal adipocyte differentiation [36]. In this study, mutation of the C/EBPß site in 3T3-L1 cells, significantly decreased the transcription activity of the *PDHB* promoter. Mixing 5’-biotin labeled C/EBPß probes with nuclear extracts of 3T3-L1 cells, led to an upshift of the band. The ChIP assay confirms the transcription factor C/EBPß to also be capable of binding to the C/EBPß binding site in the *PDHB* promoter. These results reveal the C/EBPß to be a key transcription factor for the bovine *PDHB* expression in adipocytes.

In summary, we cloned the CDS and promoter sequences of the bovine *PDHB* gene, and identified its transcription initiation sites. The amino acids and promoter sequences of bovine *PDHB* gene shared high similarity with the homologs of goat and sheep. We found the gene widely expressed with the highest level intestis fat. MYOG and C/EBPß are likely important transcription factors for the expression of the bovine *PDHB* gene in skeletal muscle cells and adipocytes, respectively. Our results provide an important basis enabling further understanding of the transcriptional regulation and biological function of the bovine *PDHB* gene.
